# ^1^H-MR Spectroscopy as a Decision-Support Tool in the Differential Diagnosis of Intracranial Lesions: A Real-World Bicentric Retrospective Cohort

**DOI:** 10.3390/diagnostics16172772

**Published:** 2026-08-28

**Authors:** Laura Maria Georgescu, Alexandru Șerbănoiu, Adrian Costache, Ioana-Andreea Cîrlig, Cristina-Mihaela Ciofiac, Nica Raluca Elena, Lucian-Mihai Florescu, Radu Tudor Ion, Ioana-Andreea Gheonea

**Affiliations:** 1 Doctoral School, University of Medicine and Pharmacy of Craiova, 200349 Craiova, Romania; laura.georgescu0112@gmail.com (L.M.G.); andreea.cirlig97@gmail.com (I.-A.C.); 2 Department of Radiology, University of Medicine and Pharmacy Carol Davila, 050474 Bucharest, Romania; 3 Department of Radiology, Emergency University Hospital Bucharest, 050098 Bucharest, Romania; 4 Pathology Department, Carol Davila University of Medicine and Pharmacy, 020021 Bucharest, Romania; 5 Department of Radiology and Medical Imaging, Emergency Clinical County Hospital of Craiova, 200642 Craiova, Romania; raluca.nica@umfcv.ro (N.R.E.);; 6 Department of Radiology and Medical Imaging, University of Medicine and Pharmacy of Craiova, 200349 Craiova, Romania; cristina.ciofiac@umfcv.ro

**Keywords:** MR spectroscopy, intracranial lesions, Cho/NAA ratio, tumor differential diagnosis, diagnostic confidence, decision support, neuro-oncology, real-world cohort

## Abstract

**Purpose**: To evaluate whether proton MR spectroscopy (^1^H-MRS) improves the differentiation of tumoral from non-tumoral intracranial lesions beyond conventional MRI, and to quantify its effect on diagnostic confidence, in a consecutive real-world bicentric cohort of diagnostically ambiguous lesions. **Methods**: This retrospective, bicentric observational study screened 122 consecutive patients who underwent brain MRI with ^1^H-MRS at two imaging centers, of whom 99 were eligible for final analysis after excluding 23 patients due to insufficient follow-up (<12 months) or incomplete reference standard. The reference standard was histopathological confirmation in 43 cases (43.4%) and structured clinico-radiological follow-up of at least 12 months in 56 cases (56.6%). The reporting radiologist’s binary diagnostic impression and confidence score were recorded before and after ^1^H-MRS. Conventional MRI alone was compared to MRI plus ^1^H-MRS using the exact McNemar test; Cho/NAA discrimination was assessed by ROC analysis with bootstrap confidence intervals, and confidence change by the Wilcoxon signed-rank test. Secondary analyses comprised an intention-to-diagnose analysis of all 99 examinations, counting non-diagnostic spectra as test failures, and an evaluable-case sensitivity analysis restricted to histopathologically confirmed patients with interpretable spectra. **Results**: Of 99 examinations, 89 (89.9%) yielded interpretable spectra. In the primary analysis, qualitative MRS interpretation demonstrated sensitivity of 76.0% (95% CI: 61.8–86.9), specificity of 94.9% (95% CI: 82.7–99.4), and accuracy of 84.3% (CI: 75.0–91.1), compared with 54.0%, 89.7% and 69.7% (59.0–79.0) for conventional MRI alone (exact McNemar *p* = 0.024). ROC analysis of the Cho/NAA ratio (*n* = 89) yielded an AUC of 0.858 (0.774–0.931); the exploratory, cohort-specific Youden-optimal cut-off was 1.41 (sensitivity 78.0%, specificity 87.2%). In the intention-to-diagnose analysis including all 99 examinations, accuracy was 78.8% (69.4–86.4). Diagnostic confidence increased significantly after ^1^H-MRS (median 1 to 2; Wilcoxon *p* < 0.001; effect size r = 0.81), with moderate or major added value in 71 of 99 examinations (71.7%). **Conclusions**: In a diagnostically heterogeneous real-world cohort, ^1^H-MRS significantly improved the accuracy achievable with conventional MRI alone and substantially increased reported diagnostic confidence, with high specificity but only moderate sensitivity. A zone-based Cho/NAA interpretative framework better reflects biological overlap than rigid binary thresholds. As this study assessed diagnostic accuracy and reported confidence rather than therapeutic decisions or patient outcomes, ^1^H-MRS should be regarded as an adjunctive decision-support modality rather than a standalone classifier in routine neuro-oncologic workflows.

## 1. Introduction

### 1.1. Clinical Background

Intracranial lesions represent a frequent diagnostic challenge in daily neuroradiology practice. Patients are commonly referred for brain magnetic resonance imaging (MRI) due to neurological symptoms or deficits, and imaging findings may reveal a wide spectrum of intracranial abnormalities with overlapping appearances. In this setting, differentiating between tumoral and non-tumoral intracranial pathology is of critical clinical importance [1,2].

This distinction carries direct management implications, as tumoral lesions may require oncologic referral or invasive sampling, while non-tumoral conditions are frequently managed conservatively. Misclassification at this stage may therefore result in unnecessary invasive procedures, delayed appropriate therapy, or inappropriate treatment strategies.

Beyond binary categorization, many clinical scenarios are characterized by diagnostic ambiguity, in which conventional imaging findings do not confidently resolve the underlying nature of the lesion. In such contexts, imaging techniques capable of reducing diagnostic uncertainty and refining interpretative confidence are of substantial clinical value.

### 1.2. Limitations of MRI

Conventional magnetic resonance imaging (MRI) represents the cornerstone of intracranial lesion evaluation, providing high-resolution anatomical and morphological information. Standard sequences, including T1-weighted, T2-weighted, fluid-attenuated inversion recovery (FLAIR), diffusion-weighted imaging (DWI), and contrast-enhanced imaging, allow assessment of lesion location, extent, signal characteristics, enhancement patterns, and associated mass effect or edema.

However, despite these capabilities, conventional MRI remains limited in its ability to reliably differentiate between tumoral and non-tumoral intracranial pathology in a significant number of cases. A wide range of non-tumoral processes, including inflammatory, demyelinating, ischemic, infectious, and post-treatment changes, may demonstrate contrast enhancement, diffusion abnormalities, and mass effect, closely mimicking tumoral lesions. Conversely, certain tumors, particularly low-grade or infiltrative neoplasms, may present with subtle or non-specific imaging features [3].

Accordingly, reliance on conventional MRI alone may leave a proportion of cases within a zone of interpretative uncertainty, highlighting the need for complementary imaging techniques capable of providing biologically informative, non-morphological data to assist clinical decision-making [4,5].

### 1.3. Principles and Known Limitations of Proton MR Spectroscopy

^1^H-MRS enables non-invasive in vivo metabolic assessment, providing biochemical information complementary to conventional MRI [6,7,8].

The most analyzed metabolites include N-acetylaspartate (NAA), a marker of neuronal integrity; choline-containing compounds (Cho), associated with cellular membrane turnover and proliferation; creatine (Cr), related to energy metabolism; as well as lactate and lipid peaks, which may reflect anaerobic metabolism or necrotic processes [9].

In tumoral lesions, increased choline levels combined with reduced NAA are frequently observed, reflecting increased cellularity and neuronal loss [10]. In contrast, non-tumoral lesions may demonstrate preserved or variably reduced NAA levels, with choline changes dependent on inflammatory or reparative activity. However, significant metabolic overlap exists among different pathological entities and interpretation remains dependent on technical quality and clinical context.

Importantly, MRS performance is influenced by several technical factors. These include voxel placement, partial volume effects, susceptibility to motion artifacts, and contamination by cerebrospinal fluid, bone marrow, or hemorrhagic products. These factors are particularly relevant in routine practice, where not all examinations yield optimal spectra and technically limited acquisitions must be integrated into clinical reasoning.

### 1.4. Previous Research

Although the diagnostic role of ^1^H-MR spectroscopy has been widely investigated, most published studies have focused on selected tumor populations, frequently enriched for histopathologically confirmed high-grade neoplasms [11,12]. Such designs provide valuable information regarding metabolic patterns and threshold behavior but may not fully reflect the heterogeneous diagnostic scenarios encountered in routine neuroimaging [13,14].

In everyday practice, imaging interpretation often involves mixed cohorts that include inflammatory, vascular, demyelinating, post-treatment, and low-grade neoplastic processes, frequently accompanied by intermediate or technically limited spectroscopic findings. In this setting, the practical contribution of MRS may extend beyond absolute diagnostic accuracy and instead relate to its ability to reduce diagnostic ambiguity and refine interpretative confidence.

Prior work by Möller–Hartmann et al. demonstrated the feasibility of ^1^H-MRS in mixed intracranial mass lesions in a single-center cohort [15]. The present study extends this framework by incorporating a prospective confidence scoring system, a predefined added value scale, and a bicentric consecutive design, enabling quantitative assessment of the interpretative contribution of MRS beyond binary diagnostic classification.

### 1.5. Rationale and Study Objective

The present study evaluates ^1^H-MR spectroscopy within the broader clinical framework of diagnostic uncertainty. Rather than focusing exclusively on classification accuracy in selected tumor populations, we assess the role of MRS as a decision-support tool in a consecutive, bicentric real-world cohort encompassing the full spectrum of intracranial lesions encountered in routine neuroradiological practice, including technically limited and metabolically indeterminate cases.

Specifically, we aim to evaluate the following:

(1) The incremental diagnostic performance of qualitative and semi-quantitative spectroscopic interpretation, compared to conventional MRI alone, in differentiating tumoral from non-tumoral lesions;
(2) The behavior of the Cho/NAA ratio within a heterogeneous population; and
(3) The impact of MRS on diagnostic confidence and ambiguity reduction in routine clinical scenarios.

## 2. Materials and Methods

### 2.1. Study Population

This retrospective, bicentric observational study screened 122 consecutive patients who underwent brain magnetic resonance imaging (MRI) with proton MR spectroscopy (^1^H-MRS) between 19 August 2021 and 20 November 2025 at two imaging centers: the Emergency University Hospital Bucharest (SUUB), a tertiary academic center and Medima Imaging Center, a private outpatient provider.

Patients were referred for neuroimaging due to neurological symptoms or deficits. Referrals followed two pathways:

(i) Direct neurological evaluation in the outpatient setting;
(ii) CT-first referral from the hospital setting when initial CT was inconclusive and MRI was requested for further characterization.

Eligibility required a conventional MRI demonstrating an intracranial lesion with a tumoral versus non-tumoral differential. ^1^H-MRS was performed specifically in cases where conventional imaging alone was insufficient for diagnostic certainty and additional metabolic characterization was considered clinically relevant.

Inclusion criteria were as follows:

- Neurological symptoms or deficits prompting brain imaging.
- Presence of intracranial lesion(s) on MRI.
- Availability of a complete MRI examination including ^1^H-MR spectroscopy.
- A documented diagnostic need for further lesion characterization with a tumor versus non-tumor differential.

Patients with standard contraindications to MRI or those unable to undergo a complete MRI examination were excluded before imaging and therefore not included in the study cohort.

Spectroscopic examinations classified as technically limited or non-diagnostic were intentionally retained and analyzed as a separate feasibility category (10 of 99, 10.1%). These were excluded from the primary diagnostic-accuracy analysis, which was therefore performed on 89 patients, and additionally handled in an intention-to-diagnose analysis in which they were counted as incorrect classifications.

Reference Standard

The final diagnosis was established by the following:

- Histopathological confirmation when available;
- Structured clinico-radiological follow-up when histopathology was not performed.

For cases without histopathological confirmation, the final diagnosis was established by structured clinico-radiological follow-up. A minimum follow-up duration of 12 months was required for inclusion.

The mean follow-up duration was 17.8 ± 4.2 months (median 18 months; range 12–26 months). Follow-up MRI examinations were typically performed at 3–6 months after baseline and subsequently at 6-month intervals. At least two follow-up MRI examinations were required to confirm lesion stability, regression, or progression consistent with the final diagnosis.

Follow-up imaging was independently reviewed by at least two experienced neuroradiologists in conjunction with clinical records. Final diagnostic classification was established by consensus, based on a multiparametric assessment incorporating lesion size trajectory, enhancement pattern evolution, diffusion characteristics, and clinical course—including treatment response, symptom evolution, and absence of oncologic intervention in cases classified as non-tumoral. Classification required concordance between imaging evolution and clinical data across a minimum of two serial examinations.

Of the 122 screened patients, 23 were excluded before final cohort assembly: 17 for insufficient longitudinal imaging or clinical follow-up (<12 months) and 6 because a definitive reference-standard diagnosis could not be established following a non-diagnostic biopsy and insufficient subsequent clinico-radiological evidence for definitive classification.

The reference standard was histopathology in 43 patients (43.4%) and structured clinico-radiological follow-up in 56 (56.6%). Verification route differed by final diagnosis: among the 57 patients with a final tumoral diagnosis, 41 (71.9%) were confirmed histopathologically and 16 (28.1%) by follow-up; among the 42 with a non-tumoral diagnosis, 2 (4.8%) had histopathology and 40 (95.2%) follow-up. This asymmetry is inherent to clinical practice, since biopsy is rarely performed once imaging and follow-up indicate a non-tumoral process.

**Figure 1:**
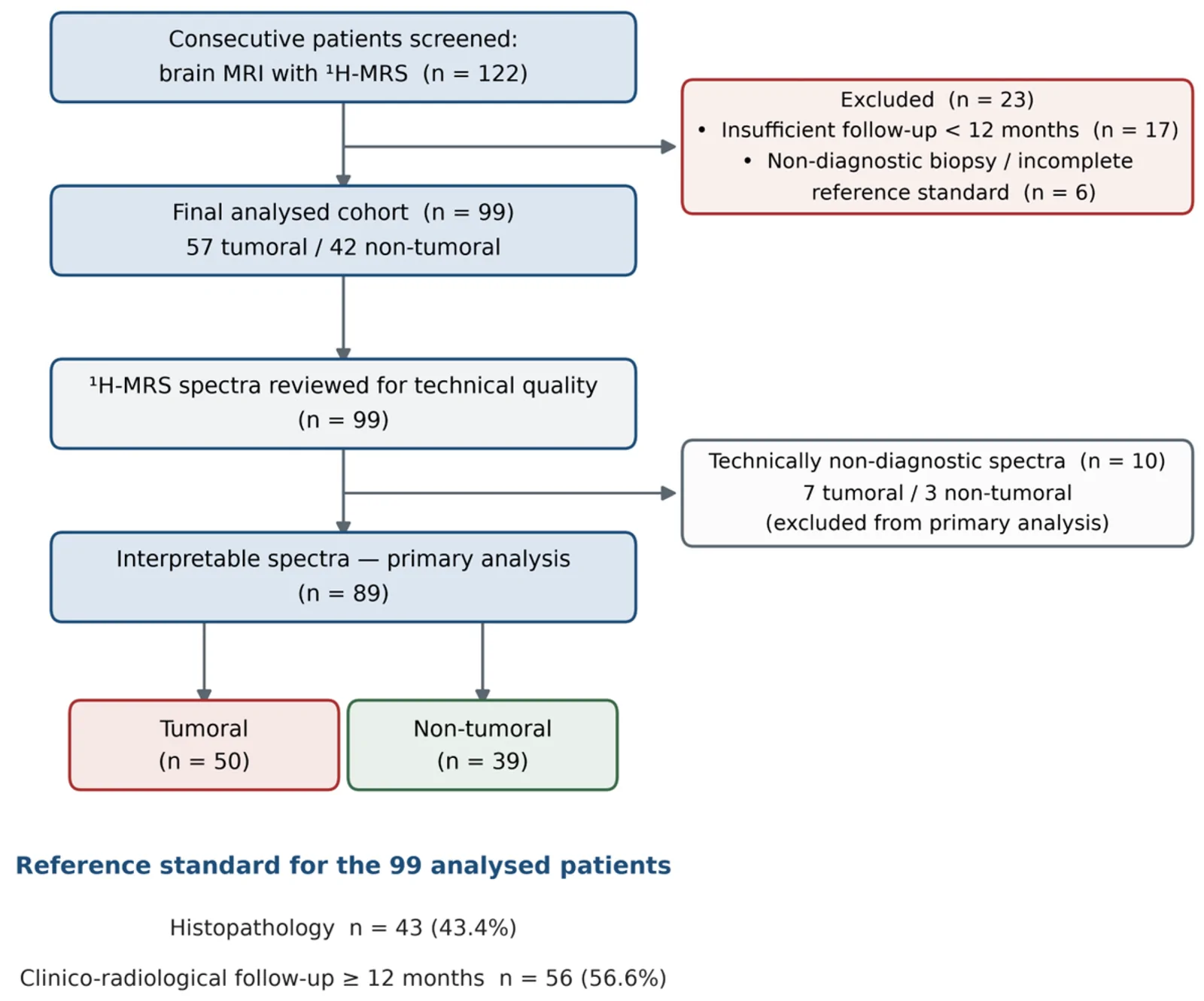
Patient recruitment and cohort assembly. Of 122 consecutive patients screened between August 2021 and November 2025, 23 were excluded before analysis (17 with follow-up shorter than 12 months and 6 with an incomplete reference standard following non-diagnostic biopsy and insufficient subsequent clinico-radiological evidence). Spectra were technically non-diagnostic in 10 of the 99 remaining patients, leaving 89 patients with interpretable spectra in the primary diagnostic-accuracy analysis.

Patient selection and cohort assembly are illustrated in Figure 1.

### 2.2. MRI Acquisition Protocol

All examinations were performed on a 3T MRI system (SIGNA™, GE Healthcare) using a standardized protocol across both centers.

The conventional MRI protocol included the following:

- T1-weighted imaging
- T2-weighted imaging
- FLAIR
- Diffusion-weighted imaging (DWI) with ADC maps
- Post-contrast T1-weighted imaging

Intravenous gadoteric acid (Dotarem; Guerbet, Villepinte, France) was administered at the standard dose of 0.1 mmol/kg body weight using a power injector at 4 mL/s, followed by saline flush. The same agent and dose were used at both centers.

Conventional MRI findings were reviewed before spectroscopy to guide voxel placement and determine the necessity of metabolic assessment.

### 2.3. MR Spectroscopy Technique

^1^H-MRS was performed during the same session. Either single-voxel spectroscopy (SVS) or multi-voxel 2D chemical shift imaging (2D-CSI) was applied depending on lesion characteristics.

Acquisition Parameters

Single-voxel spectroscopy was performed using PRESS with

- TR: 1500–2000 ms
- TE: 144 ms
- Voxel size: 1.5–8 cm^3^

TE 144 ms was selected to optimize reproducible evaluation of Cho/NAA ratios and lactate detection [16].

Multi-voxel spectroscopy was acquired using 2D-CSI with comparable TR and TE parameters.

Voxel placement was performed by trained MRI technologists and radiologists following a standardized positioning protocol applied consistently across both centers. Voxel location was determined on contrast-enhanced T1-weighted and FLAIR images, targeting the solid, metabolically active portion of the lesion—specifically the enhancing rim or non-necrotic core for tumoral lesions, and the T2-hyperintense epicenter for non-enhancing lesions. Voxel dimensions were adapted to individual lesion size to optimize signal-to-noise ratio while minimizing partial volume contamination from adjacent CSF, necrosis, hemorrhage, or normal parenchyma.

Spectral quality was visually assessed for signal-to-noise ratio, peak resolution, baseline stability, and artifact absence. Unreliable spectra were classified as non-diagnostic.

### 2.4. Image and Spectroscopic Analysis

All examinations were reviewed by board-certified neuroradiologists aware of the clinical presentation but blinded to the final diagnosis at the time of interpretation. Each case was scored by the neuroradiologist reporting it; the reader therefore varied across cases. In a subset of cases, several neuroradiologists reviewed the study together and recorded a single consensus score. Because no case was independently scored twice by design, formal interobserver agreement could not be computed.

#### 2.4.1. Conventional MRI Assessment

Lesions were initially categorized based solely on conventional MRI sequences, including contrast-enhanced imaging, according to one of the following:

(i) A predominant impression (likely tumoral or likely non-tumoral);
(ii) A differential diagnosis between two or more plausible entities, when imaging findings were inconclusive.

This classification relied on established radiological criteria such as lesion morphology, contrast enhancement pattern, diffusion restriction, surrounding edema, mass effect, and anatomical location.

For the purpose of the paired comparison with spectroscopy, the conventional-MRI assessment was reduced to a binary call: a predominant impression was taken at face value, and when a differential between two or more entities was recorded, the first-named (leading) entity was taken as the MRI call. This rule was fixed before analysis and applied uniformly.

#### 2.4.2. Spectroscopic Evaluation Criteria

Spectroscopic interpretation combined qualitative pattern recognition and semi-quantitative metabolite ratios (Cho/NAA, Cho/Cr when feasible).

Spectra were classified as

- Likely tumoral
- Likely non-tumoral
- Uncertain
- Non-diagnostic

Importantly, qualitative categorization was performed independently of subsequent statistical threshold optimization to avoid circular reasoning.

#### 2.4.3. Clinical Impact and Added Value Assessment

Added value score (0–3):

In addition to diagnostic classification, the clinical utility of MR spectroscopy was assessed using a predefined ordinal added value scale ranging from 0 to 3. The scale was prospectively defined before statistical analysis and applied systematically across all cases.

Scores were assigned as follows:

0—no contribution

1—minimal influence (no change in diagnostic direction, marginal confidence modification);

2—moderate influence (reinforcement of the leading diagnostic hypothesis);

3—major influence (change in primary diagnostic orientation).

Diagnostic confidence scale (1–3):

Diagnostic confidence was recorded before and after review of the spectroscopy on a three-point ordinal scale: 1 = low (differential unresolved, further investigation required), 2 = intermediate (a leading hypothesis favored but not conclusive), 3 = high (diagnosis considered established for management purposes). The added-value and confidence scores were assigned by the reporting neuroradiologist at the time of clinical reporting, as part of a structured institutional protocol designed to evaluate the clinical utility of ^1^H-MRS prospectively and regardless of spectroscopic outcome. Scores were recorded before the final reference diagnosis was available, ensuring independence between the utility assessment and the reference standard.

### 2.5. Statistical Analysis

The study was reported in accordance with the STARD 2015 guideline for diagnostic accuracy studies.

Statistical analyses were performed in Python 3.11 using NumPy, pandas, SciPy and scikit-learn. Confidence intervals for sensitivity, specificity, predictive values and accuracy were calculated by the Clopper–Pearson exact binomial method; confidence intervals for the area under the curve were obtained by bootstrap resampling with 10,000 replicates. Two-sided *p* < 0.05 was considered statistically significant.

Continuous variables were expressed as mean ± SD or as median with interquartile range (IQR) according to distribution, assessed with the Shapiro–Wilk test; categorical variables as counts and percentages. Cho/NAA ratios were compared between tumoral and non-tumoral lesions using the Mann–Whitney U test.

Diagnostic performance was assessed as sensitivity, specificity, positive and negative predictive values and accuracy, each with 95% confidence intervals. The reference standard was the final diagnosis established by histopathology or by structured clinico-radiological follow-up.

The primary analysis compared two index tests in the same 89 patients with interpretable spectra: conventional MRI alone, and conventional MRI combined with ^1^H-MRS. Spectra classified as uncertain were conservatively assigned to the non-tumoral category, so that the reported sensitivity is a lower bound. The two paired proportions were compared using the exact McNemar test, and the absolute gain in overall accuracy was computed as the difference between the number of cases correctly reclassified by spectroscopy and the number incorrectly reclassified, divided by the cohort size. Non-diagnostic spectra (*n* = 10) were excluded from this primary analysis and evaluated separately in an intention-to-diagnose analysis over all 99 patients, in which they were counted as incorrect.

Receiver-operating-characteristic analysis of the Cho/NAA ratio was performed with the area under the curve as the discrimination measure. A cut-off was derived by the Youden index. Because this cut-off was obtained from the same cohort in which it was evaluated, it is reported as exploratory and hypothesis-generating rather than as a validated diagnostic threshold; its optimism was quantified by leave-one-out cross-validation, and both the apparent and cross-validated accuracies are reported. No independent validation cohort was available.

The incremental contribution of lactate/lipid presence beyond the Cho/NAA ratio was assessed by comparing the classification accuracy of Cho/NAA alone with that of Cho/NAA combined with lactate/lipid status.

Changes in diagnostic confidence before and after spectroscopy were analyzed with the Wilcoxon signed-rank test for paired ordinal data, restricted to the 89 patients for whom a post-spectroscopy confidence score was recorded; the effect size r was calculated as Z/√N, where N represented the number of non-zero paired differences included in the Wilcoxon test (*n* = 66 of the 89 paired observations). Medians and interquartile ranges are reported.

No formal a priori sample size calculation was performed due to the retrospective nature of the study. All consecutive eligible cases within the study period were included.

## 3. Results

### 3.1. Study Population and Baseline Characteristics

A total of 99 consecutive eligible patients were included in the final analysis. The mean age was 51.1 ± 14.5 years (range, 19–79 years), with a nearly balanced sex distribution (52 males [52.5%], 47 females [47.5%]).

MRI examinations were performed at two centers using identical 3T systems and standardized acquisition protocols. Fifty-four patients (54.5%) were examined in the tertiary hospital setting (SUUB), and 45 (45.5%) in the outpatient imaging center (Medima). Single-voxel spectroscopy was performed in 81 cases (81.8%), and multi-voxel spectroscopy in 18 cases (18.2%).

The reference standard and verification routes are detailed in Section 2.1 and summarized in Table 1.

### 3.2. Distribution of Final Diagnoses

According to the reference standard, 57 of the 99 lesions (57.6%) were tumoral and 42 (42.4%) were non-tumoral. Spectra were technically non-diagnostic in 10 examinations (10.1%), which were excluded from the primary diagnostic-accuracy analysis; the primary analysis population therefore comprised 89 patients, of whom 50 (56.2%) had a tumoral and 39 (43.8%) a non-tumoral final diagnosis.

Among the 89 patients with interpretable spectra, tumoral diagnoses comprised glioblastoma (*n* = 15), diffuse astrocytoma (*n* = 10), oligodendroglioma (*n* = 9), anaplastic oligodendroglioma (*n* = 1), anaplastic astrocytoma (*n* = 1), metastases (*n* = 3), multinodular and vacuolating neuronal tumor (*n* = 3), meningioma (*n* = 2), primary CNS lymphoma (*n* = 2), non-Hodgkin lymphoma with CNS involvement (*n* = 1), anaplastic ependymoma (*n* = 1), medulloblastoma (*n* = 1), and hemangioblastoma (*n* = 1). Tumor classification followed the 2021 WHO classification of CNS tumors [17].

Non-tumoral diagnoses were heterogeneous and comprised demyelinating disease (multiple sclerosis *n* = 8, tumefactive demyelinating lesion *n* = 8, acute disseminated encephalomyelitis *n* = 1, osmotic demyelination syndrome *n* = 1), infectious and inflammatory conditions (encephalitis *n* = 6, progressive multifocal leukoencephalopathy *n* = 6, cerebral abscess *n* = 1, Behçet disease *n* = 1), vascular lesions (subacute infarct *n* = 3, cavernous hemangioma *n* = 1, capillary telangiectasia *n* = 1), intraparenchymal hematoma (*n* = 1) and gliosis (*n* = 1).

Among the 10 patients with non-diagnostic spectra, the final diagnosis was tumoral in 7 and non-tumoral in 3. This distribution reflects the heterogeneity typical of real-world diagnostically ambiguous intracranial lesions.

### 3.3. Feasibility and Interpretability of ^1^H-MR Spectroscopy

Spectra were interpretable in 89 of 99 examinations (89.9%). The 10 non-diagnostic examinations were attributable to motion artifact, voxel misplacement or susceptibility effects, and are accounted for in the intention-to-diagnose analysis.

Qualitative interpretation classified 40 spectra as tumoral, 37 as non-tumoral and 12 as uncertain.

### 3.4. Diagnostic Performance of Qualitative ^1^H-MRS Interpretation

In the primary analysis of the 89 patients with interpretable spectra, uncertain spectra (*n* = 12) were conservatively assigned to the non-tumoral category, so the reported sensitivity represents a lower bound. Qualitative ^1^H-MRS interpretation achieved a sensitivity of 76.0%, a specificity of 94.9% and an accuracy of 84.3%, corresponding to 38 true-positive, 37 true-negative, 2 false-positive and 12 false-negative classifications.

Conventional MRI alone, assessed in the same 89 patients, achieved a sensitivity of 54.0% and an accuracy of 69.7%. Adding ^1^H-MRS reclassified 21 patients correctly and 8 incorrectly, a significant improvement (exact McNemar test, *p* = 0.024) corresponding to an absolute gain in accuracy of 14.6 percentage points.

Of the 43 histopathologically verified patients, 41 had interpretable spectra and formed the evaluable-case histopathological subgroup; the remaining 2 had non-diagnostic spectra and were therefore included only in the intention-to-diagnose analysis.

Performance was lower in the intention-to-diagnose analysis of all 99 examinations (accuracy 78.8%) and higher in the histologically verified subgroup (*n* = 41, accuracy 87.8%) and after exclusion of uncertain spectra (*n* = 77, accuracy 89.6%), confirming that metabolically indeterminate lesions account for most false-negative results. All estimates with 95% confidence intervals are given in Table 2.

High specificity indicates that a clearly tumoral metabolic pattern strongly predicts neoplastic pathology, whereas the more modest sensitivity reflects biological overlap within intermediate metabolic profiles.

Diagnostic performance differed markedly across pathological categories (Table 3). Classification was reliable in high-grade glioma (17/18), lymphoma (3/3), demyelinating disease (17/18), infectious or inflammatory lesions (13/14) and vascular lesions (6/6), but was lower in low-grade glioma (15/19) and lowest in the heterogeneous category of other primary tumors (1/7), which comprised meningioma, multinodular and vacuolating neuronal tumor, hemangioblastoma and medulloblastoma. Median Cho/NAA in that category was 0.90 (IQR 0.71–1.02), within the range observed for non-tumoral lesions.

### 3.5. ROC Analysis and Metabolic Stratification

**Figure 2:**
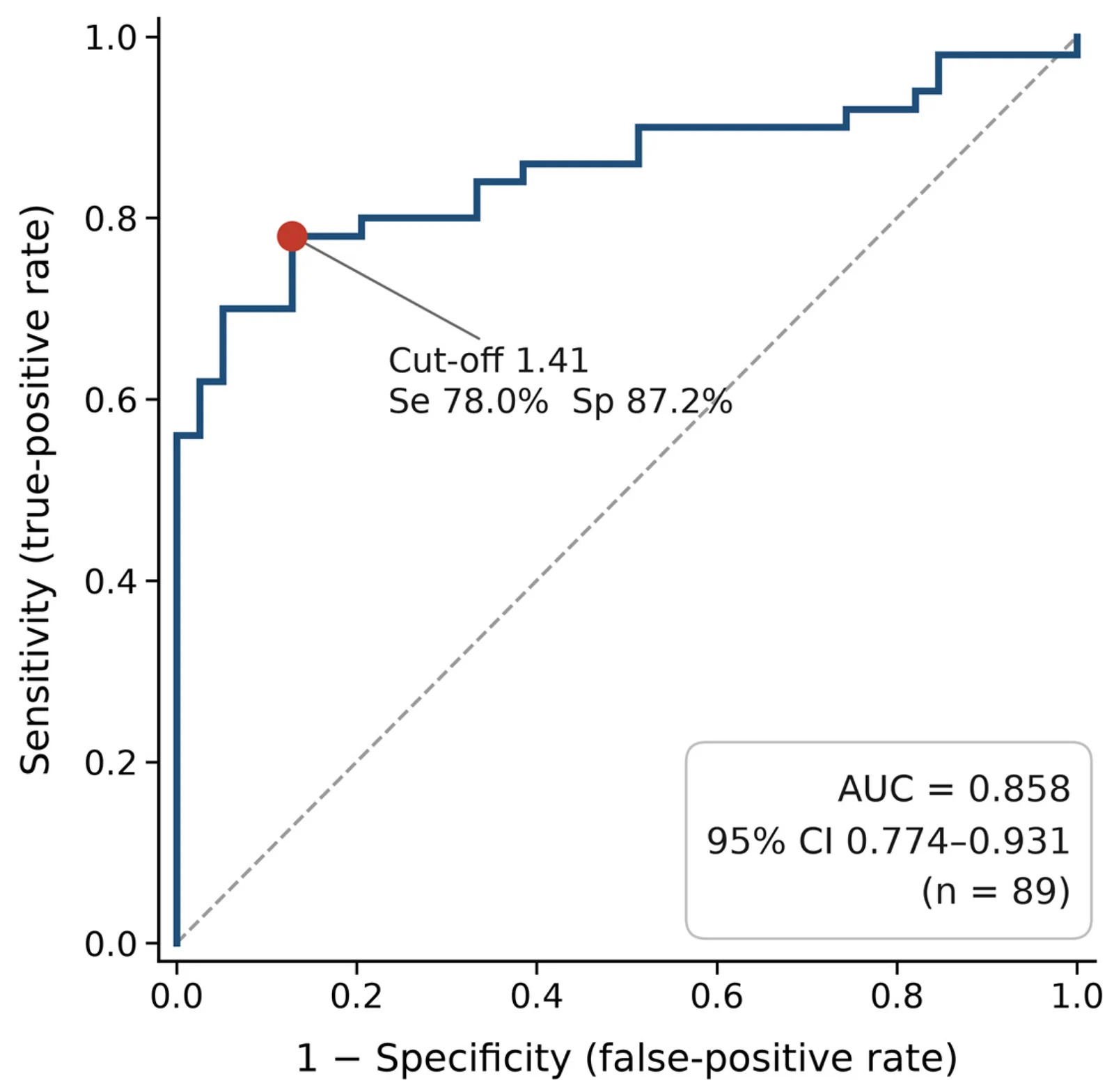
Receiver operating characteristic curve of the Cho/NAA ratio for the discrimination of tumoral from non-tumoral intracranial lesions (*n* = 89). The area under the curve is 0.858 (95% CI 0.774–0.931). The red marker indicates the exploratory Youden-optimal cut-off of 1.41 (sensitivity 78.0%, specificity 87.2%). The dashed gray line represents a non-informative classifier.

Cho/NAA ratios were higher in tumoral than in non-tumoral lesions (median 2.10, IQR 1.50–2.60 versus 0.90, IQR 0.77–1.15; Mann–Whitney U test, *p* < 0.001). ROC analysis in the 89 patients with interpretable spectra yielded an area under the curve of 0.858 (95% CI 0.774–0.931; Figure 2).

The Youden-optimal cut-off was 1.41 (sensitivity 78.0%, specificity 87.2%). Because this threshold was both derived and evaluated in the same cohort, it is reported as exploratory; leave-one-out cross-validation gave an accuracy of 77.5% against an apparent 82.0%, an optimism of 4.5 percentage points.

Adding lactate/lipid status did not significantly improve classification (accuracy 82.0% versus 83.1%; exact McNemar test, *p* = 1.00). Because lactate/lipid presence was recorded categorically rather than as a continuous measure, no comparison of ROC curves was performed.

ROC analysis parameters are summarized in Table 4.

**Figure 3:**
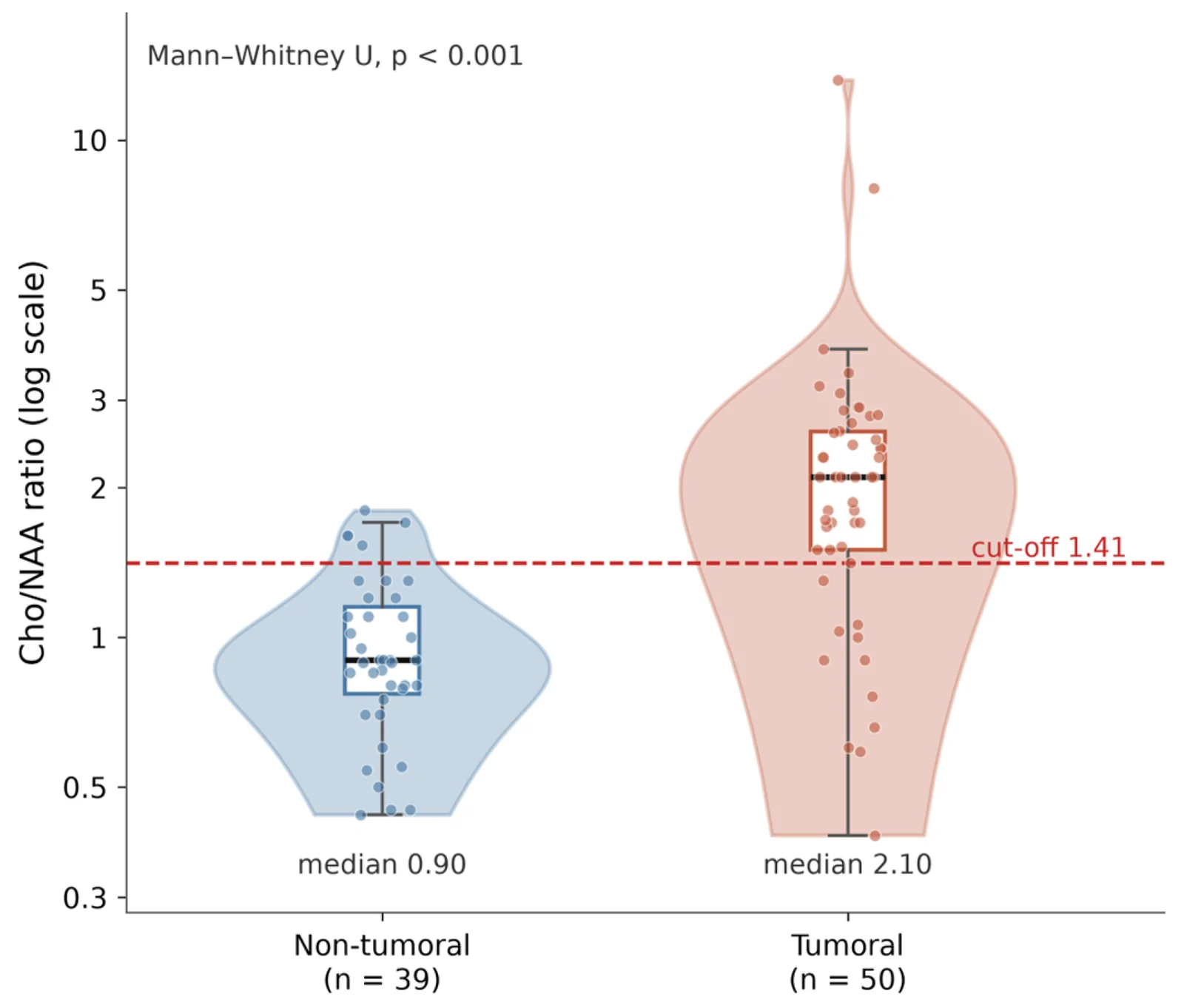
Distribution of Cho/NAA ratios by final diagnosis (*n* = 89). Violin plots show the kernel density in non-tumoral (*n* = 39) and tumoral (*n* = 50) lesions, with superimposed box plots and individual patient values. Median Cho/NAA was 0.90 (IQR 0.77–1.15) versus 2.10 (IQR 1.50–2.60) (Mann–Whitney U test, *p* < 0.001). The red dashed line marks the exploratory cut-off of 1.41. The vertical axis is logarithmic to accommodate the range of observed values.

Metabolic stratification of the 89 interpretable examinations showed three interpretative zones: Cho/NAA < 1 (*n* = 31) was predominantly non-tumoral (24/31, 77.4%); Cho/NAA of 1–2 (*n* = 31) formed a gray zone with near-equal representation of both categories (16 tumoral, 15 non-tumoral); and Cho/NAA > 2 (*n* = 27) was exclusively tumoral (27/27, 100%). These exploratory proportions require external validation, but the zone-based distribution reflects clinical ambiguity better than a rigid binary threshold (Figure 3).

### 3.6. Clinical Impact and Added Diagnostic Value of ^1^H-MRS

Diagnostic confidence was compared before and after spectroscopy in the 89 patients with an assessable post-spectroscopy score; no such score was recorded for the 10 technically non-diagnostic examinations, which were handled separately in the intention-to-diagnose analysis.

Median confidence rose from 1 (IQR 1–2) to 2 (IQR 2–3) (Wilcoxon signed-rank test, *p* < 0.001; effect size r = 0.81), increasing in 61 patients (68.5%), remaining unchanged in 23 (25.8%) and decreasing in 5 (5.6%).

**Figure 4:**
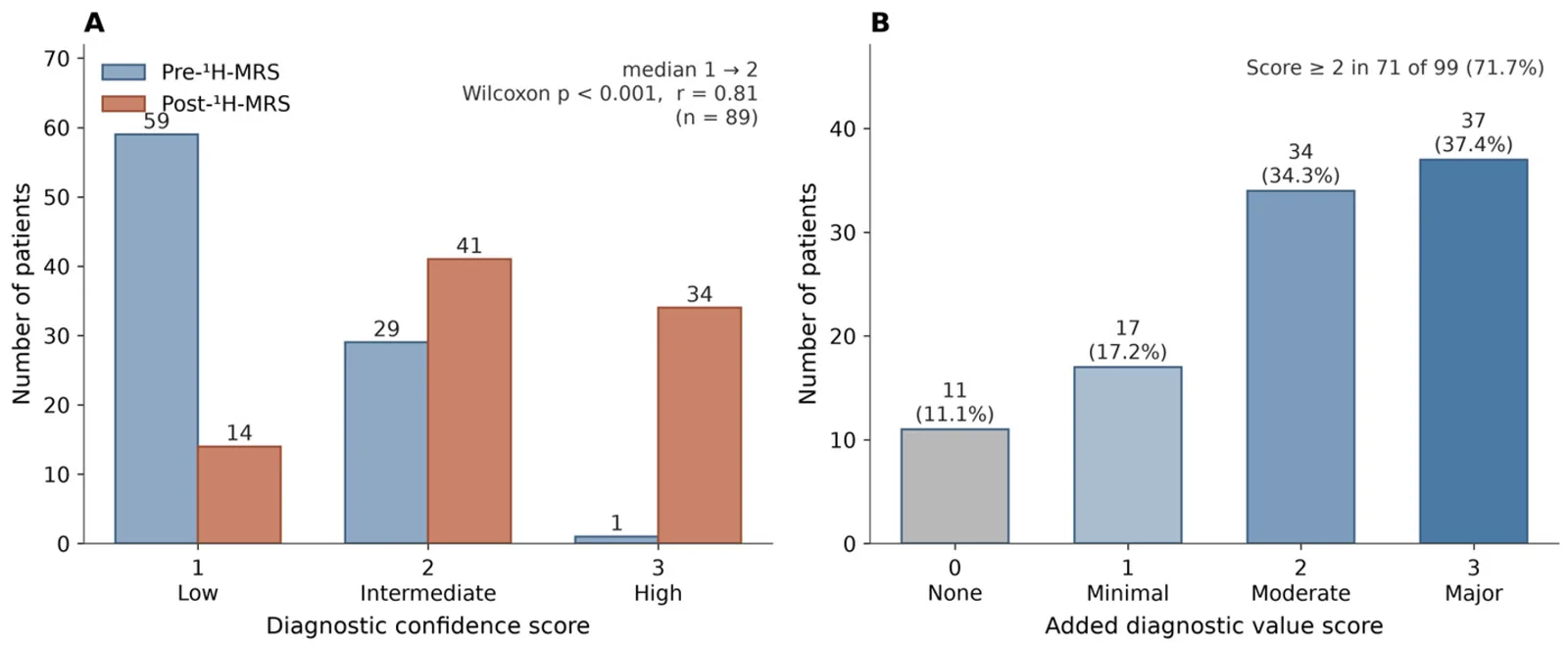
Clinical impact of ^1^H-MR spectroscopy. (**A**) Diagnostic confidence before and after spectroscopy in the 89 patients with an assessable post-spectroscopy score; median rose from 1 (IQR 1–2) to 2 (IQR 2–3) (Wilcoxon signed-rank test, *p* < 0.001; r = 0.81). (**B**) Added diagnostic value across all 99 examinations; moderate or major added value in 71 of 99 (71.7%).

Added diagnostic value, scored across all 99 examinations, was major in 37 cases (37.4%), moderate in 34 (34.3%), minimal in 17 (17.2%) and absent in 11 (11.1%); moderate or major added value was therefore recorded in 71 of 99 examinations (71.7%). Within the Cho/NAA gray zone (*n* = 31), confidence increased in 16 patients (51.6%) and moderate or major added value was recorded in 20 (64.5%), indicating that spectroscopy frequently clarified even metabolically overlapping lesions (Table 5, Figure 4).

The increase in reported confidence was accompanied by a corresponding increase in diagnostic correctness (Table 6). Before spectroscopy, accuracy was 62.7% (95% CI 50.0–73.9) at low confidence (*n* = 59) and 82.8% (65.5–92.4) at intermediate confidence (*n* = 29); only one examination was reported with high confidence. After spectroscopy, accuracy was 57.1% (32.6–78.6) at low confidence (*n* = 14), 87.8% (74.5–94.7) at intermediate confidence (*n* = 41) and 91.2% (77.0–97.0) at high confidence (*n* = 34). The post-spectroscopy confidence scale was therefore monotonically ordered with respect to accuracy, whereas the pre-spectroscopy scale was compressed into its two lowest levels; examinations that remained of low confidence after spectroscopy retained no discriminative value.

## 4. Discussion

### 4.1. Diagnostic Performance Within a Clinically Ambiguous Cohort

**Figure 5:**
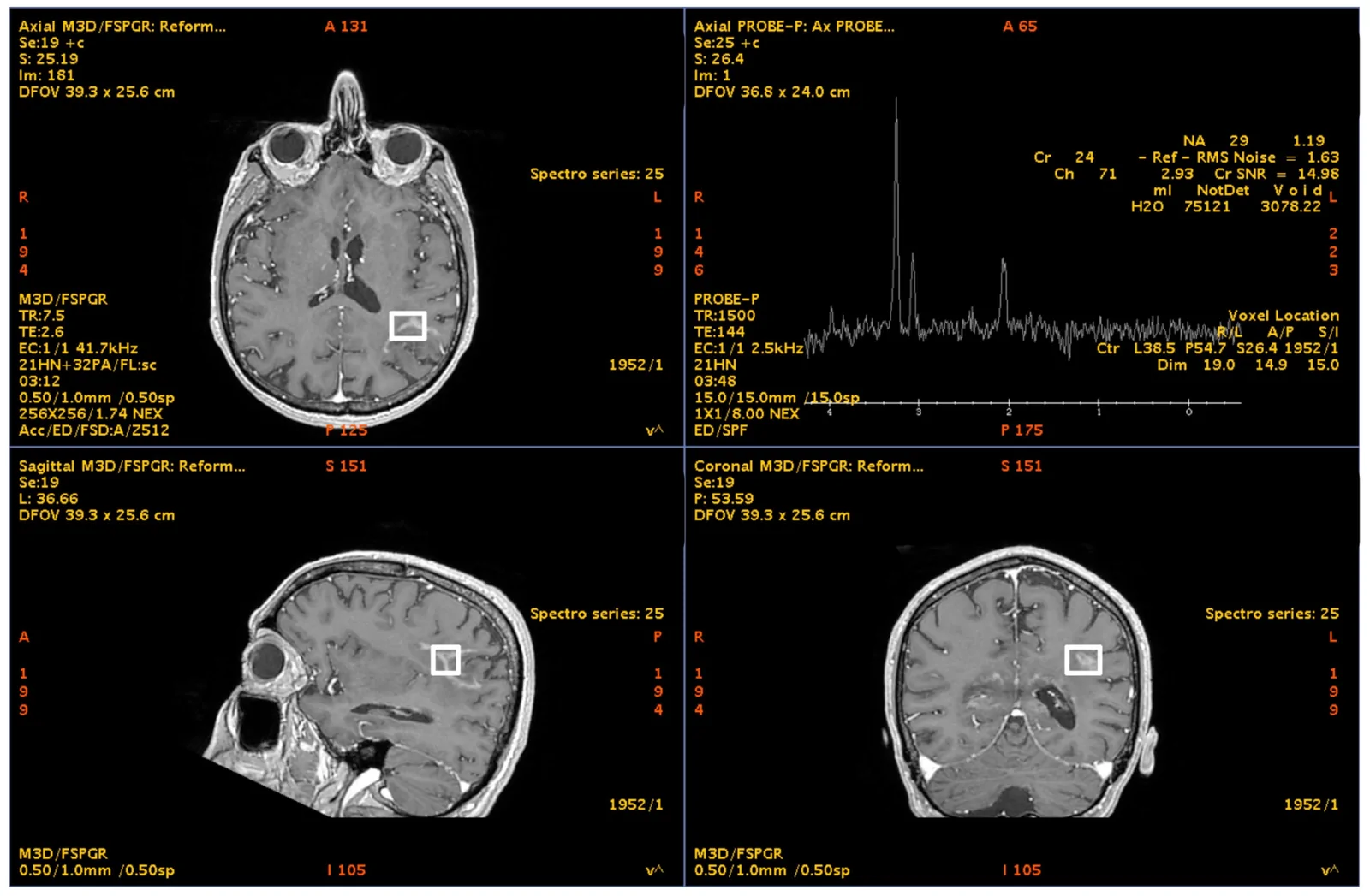
Multifocal glioblastoma with atypical radiologic presentation. Post-contrast T1-weighted MRI demonstrates multiple intracranial enhancing lesions involving the corpus callosum and left parietal region, with additional linear frontoparietal enhancement, raising differential considerations including inflammatory, infectious, or neoplastic processes. Restricted diffusion and classic ring-enhancement morphology were not prominent. Single-voxel ^1^H-MRS positioned within the parietal lesion demonstrates elevated choline and reduced N-acetylaspartate, resulting in an increased Cho/NAA ratio (2.4) consistent with a high-grade neoplastic metabolic pattern. Due to the atypical imaging appearance and biopsy difficulty, spectroscopy contributed to reinforcing the suspicion of high-grade tumor. Histopathological examination confirmed glioblastoma.

In this bicentric cohort enriched for diagnostically indeterminate intracranial lesions, ^1^H-MR spectroscopy demonstrated high specificity and moderate sensitivity for differentiating tumoral from non-tumoral pathology. Importantly, its principal contribution in this setting was not binary tumor detection but the structured reduction in diagnostic uncertainty. The incremental contribution of spectroscopy was quantified against conventional MRI in the same patients. Conventional MRI alone correctly classified 69.7% of lesions, with a sensitivity of 54.0%; adding ^1^H-MRS raised accuracy to 84.3% and sensitivity to 76.0% (exact McNemar test, *p* = 0.024). Spectroscopy reclassified 21 patients correctly and 8 incorrectly, raising overall accuracy by 14.6 percentage points. Notably, 17 of the 21 correctly reclassified lesions were tumors that conventional imaging had favored as non-tumoral, indicating that the principal gain lay in recognizing neoplasia in lesions whose morphology was misleading. A representative example of a metabolically aggressive lesion correctly classified as tumoral by ^1^H-MR spectroscopy is shown in Figure 5.

The inclusion of cases specifically referred for ^1^H-MRS introduces an inherent enrichment for diagnostically ambiguous presentations, which may limit generalizability to unselected neuroimaging populations. However, this design reflects the intended clinical application of spectroscopy as a problem-solving modality rather than a screening tool.

**Figure 6:**
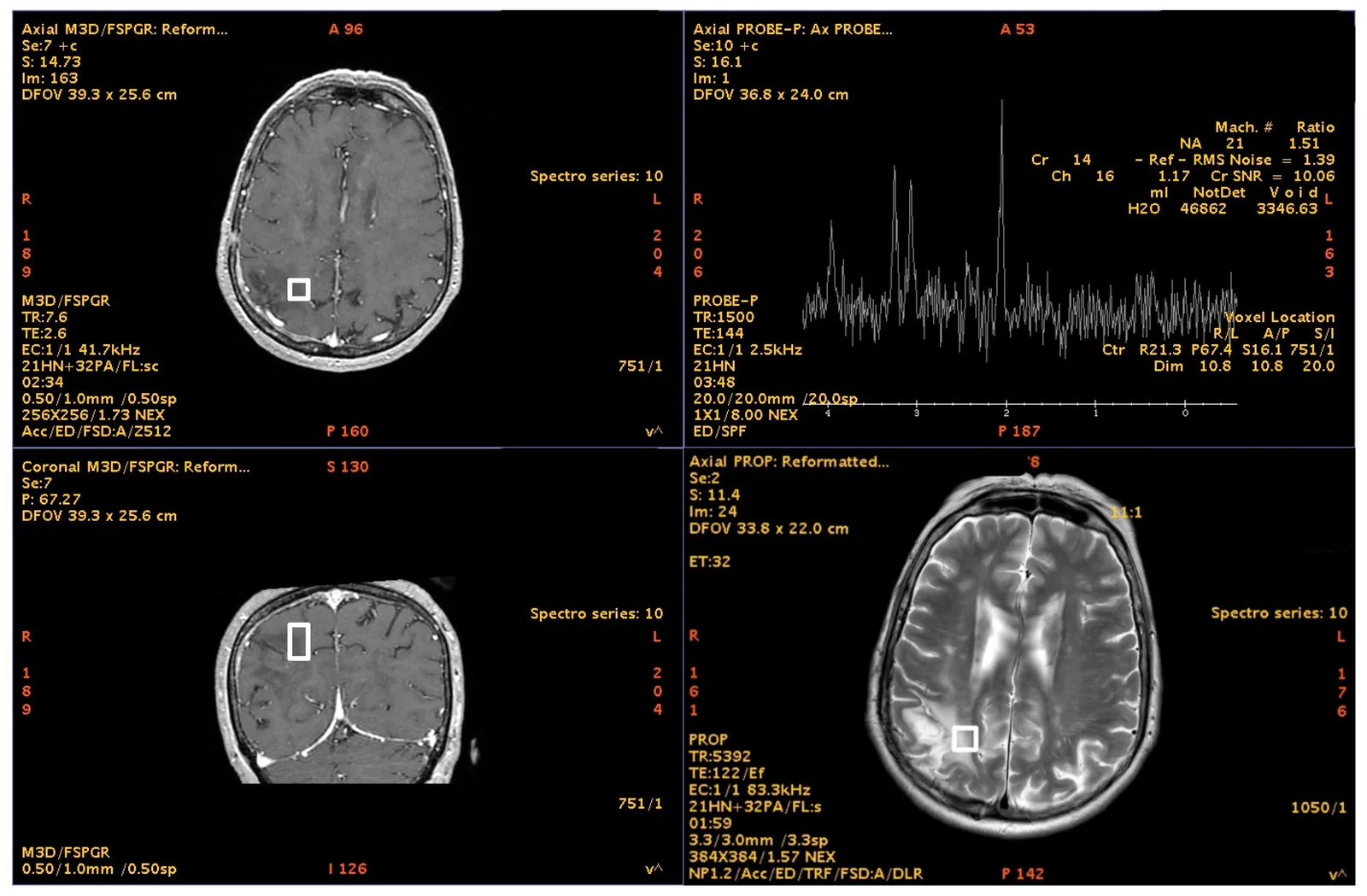
Infectious tumor mimic in an immunocompromised patient with known B-cell non-Hodgkin lymphoma. Axial and coronal post-contrast T1-weighted images demonstrate a left parietal cortical–subcortical enhancing lesion with surrounding edema, raising suspicion for secondary CNS lymphoma involvement. Axial T2-weighted imaging shows corresponding hyperintensity. Single-voxel ^1^H-MR spectroscopy (PRESS, TE = 144 ms) positioned within the enhancing region demonstrates preserved N-acetylaspartate and only mildly elevated choline, with a Cho/NAA ratio of approximately 0.76, falling within the non-neoplastic metabolic range defined in this study. Based on clinico-radiological follow-up according to the predefined protocol (minimum 12 months; serial MRI at 3–6 month intervals), the lesion resolved following targeted antibiotic therapy, and the final diagnosis was acute pneumococcal meningoencephalitis.

Unlike studies restricted to histologically confirmed neoplasms, our population included inflammatory, demyelinating, vascular, and post-treatment entities that frequently mimic neoplasia on conventional MRI [18,19]. An illustrative example of an infectious tumor mimic in an immunocompromised patient is presented in Figure 6.

Within such a spectrum, moderate sensitivity reflects biological overlap rather than technical insufficiency.

The pattern of misclassification supports this interpretation. All 12 false-negative results occurred in tumors with low or intermediate metabolic activity—multinodular and vacuolating neuronal tumor (*n* = 3), oligodendroglioma (*n* = 3), meningioma (*n* = 2), diffuse astrocytoma, glioblastoma, hemangioblastoma and metastasis (*n* = 1 each)—whereas both false-positive results occurred in non-tumoral entities with high membrane turnover, namely progressive multifocal leukoencephalopathy and an active demyelinating lesion. Accuracy accordingly rose from 84.3% to 89.6% when metabolically indeterminate spectra were excluded, confirming that the limits of the technique lie in the metabolic overlap between low-grade neoplasia and active non-tumoral processes rather than in acquisition quality.

### 4.2. Cohort-Dependent Interpretation of the Cho/NAA Threshold

The ROC-derived Cho/NAA threshold of 1.41 is lower than values commonly reported in glioma-focused cohorts [20,21,22]. Threshold behavior, however, is intrinsically population-dependent. In tumor-enriched datasets, metabolic separation is amplified by pronounced membrane turnover and neuronal loss. In contrast, inclusion of inflammatory and reactive conditions shifts the optimal discrimination boundary downward.

Accordingly, the 1.41 cut-off should be interpreted as a statistical equilibrium point within this biologically mixed population rather than a universal metabolic constant. Its cohort dependence is quantifiable: leave-one-out cross-validation reduced accuracy from an apparent 82.0% to 77.5%, an optimism of 4.5 percentage points, which is the magnitude of overfitting expected when a threshold is both derived and tested in a single sample of this size. This reinforces the need to contextualize quantitative spectroscopic thresholds within the diagnostic spectrum under investigation.

### 4.3. The Limited Incremental Role of Lactate in Tumor Detection

Although classically associated with necrosis and aggressive tumor biology, the addition of lactate/lipid status to the Cho/NAA ratio did not significantly improve classification accuracy (82.0% versus 83.1%; exact McNemar test, *p* = 1.00). Because lactate and lipid resonances were recorded categorically as present or absent rather than quantified, this comparison was made on classification accuracy and no comparison of ROC curves was possible; the finding therefore indicates that lactate presence carries little independent information for the tumoral versus non-tumoral distinction, and is compatible with the view that it reflects aggressiveness rather than tumor presence per se. In the tumor-versus-non-tumor framework, Cho/NAA remains the dominant discriminator, whereas lactate may have greater relevance in grading paradigms.

### 4.4. The Metabolic Gray Zone and Zone-Based Interpretation

**Figure 7:**
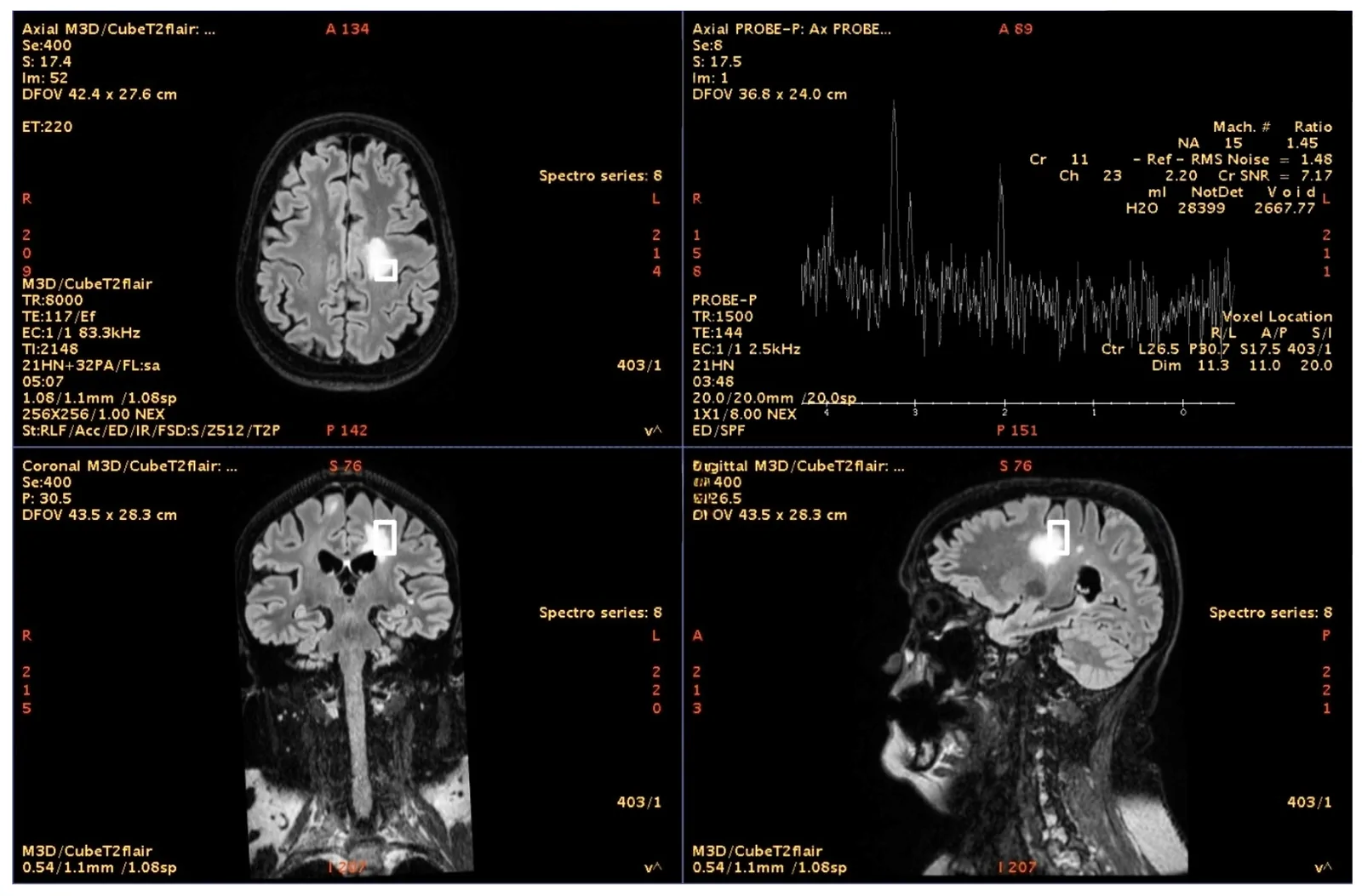
Gray-zone metabolic overlap in a tumefactive demyelinating lesion. Axial, coronal, and sagittal FLAIR images demonstrate a left frontal white matter lesion with mass effect and incomplete ring-like enhancement, raising differential considerations of low-grade glioma versus inflammatory pathology. Single-voxel ^1^H-MR spectroscopy (PRESS, TE = 144 ms) positioned within the lesion demonstrates moderately elevated choline and partially reduced N-acetylaspartate, with a Cho/NAA ratio of approximately 1.45, falling within the predefined metabolic gray zone (1–2). Given the indeterminate metabolic profile, follow-up imaging was recommended. Subsequent MRI demonstrated lesion evolution consistent with a tumefactive demyelinating lesion, and the patient was later diagnosed with multiple sclerosis.

The Cho/NAA range of 1–2 represents a true metabolic overlap region. In such cases, spectroscopy may appropriately maintain diagnostic uncertainty and prompt structured follow-up rather than premature classification. In this cohort the gray zone was substantial and genuinely balanced: 31 of 89 lesions (34.8%) had a Cho/NAA ratio between 1 and 2, of which 16 proved tumoral and 15 non-tumoral. Spectroscopy nevertheless retained interpretative value within this band, with diagnostic confidence increasing in 16 of these 31 patients (51.6%) and moderate or major added value recorded in 20 (64.5%). At the extremes the separation was clear-cut: none of the 27 lesions with Cho/NAA above 2 was non-tumoral, and 24 of 31 lesions below 1 (77.4%) were non-tumoral. A representative gray-zone case is illustrated in Figure 7.

Reactive gliosis, inflammatory infiltration, moderate membrane turnover, and partial neuronal loss may produce similar spectroscopic signatures in both inflammatory and low-grade neoplastic processes [23,24]. Rather than indicating methodological weakness, this overlap reflects intrinsic biological convergence.

The per-category data show that this overlap is not symmetric. It is populated from the non-tumoral side by a minority of highly active demyelinating and infectious lesions, in which inflammatory infiltration and membrane turnover elevate choline, and from the tumoral side by neoplasms with low proliferative activity, particularly meningioma, multinodular and vacuolating neuronal tumor and hemangioblastoma (Table 3). The clinical corollary is that the negative predictive value of ^1^H-MRS cannot be relied upon in low-proliferation neoplasms: a normal spectrum in a morphologically suspicious lesion does not exclude a tumor, and within the intermediate Cho/NAA zone spectroscopy is more appropriately reported as non-contributory than forced into a binary classification.

This zone-based behavior is consistent with previously reported Cho/NAA thresholds distinguishing high-grade gliomas from tumefactive demyelinating lesions [25].

### 4.5. Clinical Relevance: Beyond Accuracy Metrics

The principal contribution of ^1^H-MRS in this cohort lies in the measurable reduction in diagnostic ambiguity rather than the maximization of binary classification accuracy.

The increase in diagnostic confidence was both statistically significant and substantial in magnitude, rising from a median of 1 to 2 with a large effect size (Wilcoxon signed-rank test, *p* < 0.001; r = 0.81). Confidence increased in 61 of 89 patients (68.5%) and decreased in only 5 (5.6%), supporting the role of ^1^H-MRS as a tool for ambiguity reduction rather than binary classification, with clinically meaningful clarification observed even within the metabolic gray zone.

It is important to emphasize that this study evaluated interpretative impact rather than direct therapeutic modification. Changes in biopsy decisions or treatment strategies were not systematically quantified due to the retrospective design. Therefore, the contribution of spectroscopy should be understood primarily as interpretative support within the diagnostic workflow.

### 4.6. Limitations

The retrospective design and partial reliance on clinico-radiological follow-up represent inherent constraints. Despite a minimum 12-month follow-up requirement, classification bias cannot be entirely excluded, although final diagnoses were established by consensus of at least two neuroradiologists based on multiparametric imaging and clinical criteria.

The reference standard was not applied uniformly across the two diagnostic groups. Among the 57 patients with a final tumoral diagnosis, 41 (71.9%) were verified histopathologically, whereas among the 42 patients with a non-tumoral diagnosis only 2 (4.8%) underwent biopsy and 40 (95.2%) were classified by structured clinico-radiological follow-up. This differential verification is unavoidable in clinical practice, since biopsy is not justified once imaging and follow-up indicate a non-tumoral process, but it means that the non-tumoral reference category rests partly on imaging stability. Specificity may therefore be overestimated relative to a design in which all lesions were histologically verified, and the specificity of 94.9% should be read with this in mind.

Confidence and added value assessments were assigned by multiple reporting neuroradiologists across the study period, reflecting real-world clinical reporting practice. No formal interobserver agreement analysis was performed across readers, which limits the formal assessment of reproducibility of these metrics. In addition, the optimal Cho/NAA cut-off of 1.41 was derived and evaluated within the same cohort, which may lead to an optimistic estimate of its discriminative performance; leave-one-out cross-validation indicated an optimism of 4.5 percentage points (apparent accuracy 82.0% versus cross-validated 77.5%). External validation in an independent sample is warranted.

Additionally, spectroscopy remains operator-dependent, and voxel placement may influence quantitative ratios.

The wide voxel size range (1.5–8 cm^3^) used across examinations may have introduced partial volume effects, potentially influencing Cho/NAA quantification particularly in smaller lesions or those adjacent to CSF spaces.

The zone-based metabolic thresholds (Cho/NAA <1 and >2) were derived from the same cohort used for primary ROC analysis and should be regarded as exploratory. External validation in independent cohorts is required prior to clinical implementation.

Finally, biological overlap within intermediate metabolic ranges cannot be fully resolved by spectroscopy alone.

### 4.7. Future Directions

Future studies should pursue prospective multicenter validation of zone-based Cho/NAA interpretation and evaluate the integration of ^1^H-MRS within multiparametric frameworks combining diffusion and perfusion metrics [26]. Systematic assessment of downstream clinical impact, including biopsy planning and therapeutic decision modification, would further define the real-world utility of spectroscopy beyond interpretative confidence.

## 5. Conclusions

^1^H-MR spectroscopy demonstrated high specificity and moderate sensitivity for differentiating tumoral from non-tumoral pathology in this diagnostically heterogeneous cohort, and significantly improved on the accuracy achievable with conventional MRI alone in the same patients (84.3% versus 69.7%; *p* = 0.024). The Cho/NAA ratio emerged as the principal metabolic discriminator, with an ROC-derived threshold of 1.41 reflecting cohort composition rather than a universal biological constant.

Lactate/lipid peak integration did not significantly improve tumor detection performance, supporting the concept that lactate may be more informative for tumor aggressiveness than for tumor presence.

A zone-based metabolic framework provided clinically meaningful stratification beyond rigid binary thresholds, particularly within intermediate Cho/NAA ranges where biological overlap is intrinsic.

Most importantly, spectroscopy significantly increased diagnostic confidence in ambiguous cases, reinforcing its role as an adjunctive decision-support modality within routine neuro-oncologic workflows.

^1^H-MR spectroscopy should therefore be regarded not as a standalone classifier, but as a biologically informative tool capable of reducing diagnostic ambiguity in heterogeneous real-world settings.

## Figures and Tables

**Table 1:** Baseline characteristics of the analyzed cohort (*n* = 99). Values are means ± standard deviation or counts with percentages. SVS = single-voxel spectroscopy; CSI = chemical shift imaging; F/U = follow-up.

Characteristic	Value
Total Analyzed Cohort	99 patients
Mean Age (years)	51.1 ± 14.5
Sex (Male/Female)	52 (52.5%)/47 (47.5%)
Center: Tertiary Hospital (SUUB)	54 (54.5%)
Center: Outpatient Clinic (Medima)	45 (45.5%)
Technique: Single-voxel (SVS)	81 (81.8%)
Technique: Multi-voxel (CSI)	18 (18.2%)
Reference: Histopathology	43 (43.4%)
Reference: Clinico-radiological F/U	56 (56.6%)

**Table 2:** Diagnostic performance of conventional MRI alone and of conventional MRI combined with ^1^H-MR spectroscopy. Values are percentages with 95% Clopper–Pearson exact confidence intervals. ITD = intention-to-diagnose analysis of all 99 examinations, in which non-diagnostic spectra were counted as incorrect. In the histologically verified subgroup only 2 patients had a non-tumoral final diagnosis, so specificity and negative predictive value are not reported. Exact McNemar test comparing conventional MRI alone with MRI + ^1^H-MRS in the same 89 patients: *p* = 0.024; absolute gain in accuracy +14.6 percentage points.

Metric	MRI Alone (*n* = 89)	MRI + ^1^H-MRS (*n* = 89)	ITD (*n* = 99)	Histology, Interpretable MRS (*n* = 41)	Uncertain Excl. (*n* = 77)
Sensitivity	54.0 (39.3–68.2)	76.0 (61.8–86.9)	66.7 (52.9–78.6)	87.2 (72.6–95.7)	86.4 (72.6–94.8)
Specificity	89.7 (75.8–97.1)	94.9 (82.7–99.4)	95.2 (83.8–99.4)	—	93.9 (79.8–99.3)
PPV	87.1 (70.2–96.4)	95.0 (83.1–99.4)	95.0 (83.1–99.4)	100 (89.7–100)	95.0 (83.1–99.4)
NPV	60.3 (46.6–73.0)	75.5 (61.1–86.7)	67.8 (54.4–79.4)	—	83.8 (68.0–93.8)
Accuracy	69.7 (59.0–79.0)	84.3 (75.0–91.1)	78.8 (69.4–86.4)	87.8 (73.8–95.9)	89.6 (80.6–95.4)

**Table 3:** Cho/NAA distribution and ^1^H-MRS classification performance by pathological category (*n* = 89 interpretable examinations). Values are medians with interquartile range. “Uncertain” denotes spectra reported as metabolically indeterminate, which were counted as non-tumoral in the primary analysis. Categories with fewer than five patients are shown for completeness and are descriptive only.

Pathological Category	n	Cho/NAA, Median (IQR)	Correctly Classified, n (%)	FN	FP	Uncertain
High-grade glioma	18	2.42 (2.10–2.80)	17 (94.4)	1	0	0
Low-grade glioma	19	1.72 (1.58–2.20)	15 (78.9)	4	0	4
Lymphoma	3	3.10 (2.70–3.45)	3 (100)	0	0	0
Metastasis	3	2.10 (1.81–2.10)	2 (66.7)	1	0	1
Other primary tumor	7	0.90 (0.71–1.02)	1 (14.3)	6	0	1
Demyelinating	18	0.90 (0.72–1.06)	17 (94.4)	0	1	3
Infectious/inflammatory	14	0.94 (0.80–1.18)	13 (92.9)	0	1	2
Vascular	6	0.86 (0.78–0.98)	6 (100)	0	0	1
Other non-tumoral	1	1.30	1 (100)	0	0	0
Total	89	—	75 (84.3)	12	2	12

**Table 4:** ROC analysis of the Cho/NAA ratio (*n* = 89). The confidence interval for the area under the curve was obtained by bootstrap resampling with 10,000 replicates.

Parameter	Value
Area under the ROC curve	0.858 (95% CI 0.774–0.931)
Youden-optimal cut-off (exploratory)	1.41
Sensitivity/specificity at cut-off	78.0%/87.2%
Apparent accuracy	82.0%
Leave-one-out cross-validated accuracy	77.5% (optimism 4.5 points)
Median Cho/NAA, tumoral	2.10 (IQR 1.50–2.60)
Median Cho/NAA, non-tumoral	0.90 (IQR 0.77–1.15)
Group comparison (Mann–Whitney U)	*p* < 0.001
Accuracy: Cho/NAA alone vs. + lactate/lipid	82.0% vs. 83.1% (McNemar *p* = 1.00)

**Table 5:** Clinical impact of ^1^H-MR spectroscopy. Confidence analysis is restricted to the 89 patients with an assessable post-spectroscopy score; added-value scores were assigned in all 99 examinations.

Clinical Metric	Result
Pre-/post-^1^H-MRS confidence, median (IQR)	1 (1–2) → 2 (2–3)
Wilcoxon signed-rank test	*p* < 0.001
Effect size (r)	0.81
Confidence increased/unchanged/decreased	61 (68.5%)/23 (25.8%)/5 (5.6%)
Added value: major (3)	37/99 (37.4%)
Added value: moderate (2)	34/99 (34.3%)
Added value: minimal (1)	17/99 (17.2%)
Added value: none (0)	11/99 (11.1%)
Moderate or major added value (≥ 2)	71/99 (71.7%)

**Table 6:** Diagnostic accuracy at each level of reported diagnostic confidence, before and after ^1^H-MR spectroscopy (*n* = 89). Confidence was recorded on a predefined three-point scale (1 = low, 2 = intermediate, 3 = high). Accuracy denotes agreement of the radiological classification at that time point with the reference standard. Confidence intervals are Wilson score intervals.

Time Point	Confidence Level	n	Correct, n	Accuracy, % (95% CI)
Pre-^1^H-MRS	1 (low)	59	37	62.7 (50.0–73.9)
	2 (intermediate)	29	24	82.8 (65.5–92.4)
	3 (high)	1	1	100 (20.7–100)
Post-^1^H-MRS	1 (low)	14	8	57.1 (32.6–78.6)
	2 (intermediate)	41	36	87.8 (74.5–94.7)
	3 (high)	34	31	91.2 (77.0–97.0)

## Data Availability

The anonymized patient-level dataset supporting all reported analyses is provided as Supplementary Table S1. Any further data are available from the corresponding author upon reasonable request.

## Supplementary Materials

The following supporting information can be downloaded at https://www.mdpi.com/article/10.3390/diagnostics16172772/s1, Table S1. Anonymized patient dataset including demographic data, lesion characteristics, MRS parameters, and final diagnoses for all 99 included patients.
